# The impact of the union of lesser trochanter fragments after intramedullary fixation of trochanteric femoral fractures: an X-ray based study

**DOI:** 10.1186/s12891-022-05534-z

**Published:** 2022-06-22

**Authors:** Jiongming You, Feng Wang, Feng Li, Yinsheng Wu, Yong Wang, Zifei Chen

**Affiliations:** 1 Department of Orthopedic, Wenzhou Hospital of Integrated Traditional Chinese and Western Medicine, WenZhou, 325000 ZheJiang China; 2 Department of Operation Room, The Second Affiliated Hospital and Yuying Children’s Hospital of WenZhou Medical University, WenZhou, 325000 ZheJiang China

**Keywords:** Hip fractures, Intertrochanteric fractures, Lesser trochanter, Fractures, Ununited, Hip displacement, Fracture fixation, Intramedullary

## Abstract

**Background:**

Displacement of the lesser trochanter (LT) is not uncommon after managing intertrochanteric femoral fractures and the influence of nonunion of the LT-fragment on clinical outcomes remains controversial. This study aimed to investigate the relationship between the displacement distance and union of the LT-fragment and evaluate the influence of LT-fragment nonunion on hip function and complications.

**Methods:**

This retrospective study included patients with intertrochanteric fractures and displaced LT treated with intramedullary fixation at Wenzhou Hospital of Integrated Traditional Chinese and Western Medicine from June 2015 to July 2017. The patients were grouped as union and nonunion of the LT-fragment at 1 year. The LT-fragment displacement distance of LT was measured by the anterior–posterior radiographs.

**Results:**

Thirty-one and 22 patients showed union and nonunion at 1 year, respectively. The nonunion group had a higher postoperative complication rate than the union group (59% vs. 29%, *P* = 0.047), especially mechanical complications (45% vs. 6%, *P* = 0.001). There was no significant difference in hip function between the two groups (*P* > 0.05). The receiver operating characteristic (ROC) curve revealed an area under the curve of 0.933 of displacement ratio. Patients with a displacement ratio > 0.35 were more likely to have nonunion of the LT-fragment.

**Conclusions:**

The displacement ratio might be a reliable predictor of LT-fragment union. The incidence of postoperative complications might increase with LT-fragment nonunion.

## Background

Hip fractures commonly occur after a low-energy trauma in aged people with osteoporosis and are strongly associated with high morbidity and mortality [1–3]. Moreover, hip fractures represent a substantial healthcare burden for society [4]. Intertrochanteric fractures are the most reported type of hip fracture [2, 3, 5]. Therefore, surgical treatment is generally indicated, and the fixation is established either through extramedullary or intramedullary devices. Dynamic hip screw (DHS) has been known to be the best treatment option for simple and stable trochanteric fractures. And intramedullary nails are widely used worldwide for unstable trochanteric fractures [6–8].

The displacement of the lesser trochanter (LT) is not uncommon in intertrochanteric fractures. Indeed, the LT is not usually fixed when intramedullary fixation is performed to treat intertrochanteric fractures. Although some studies suggested that the integrity of the LT does not affect the surgical outcomes [9], several studies revealed that the LT-fragment is crucial to the stability of intertrochanteric fractures, especially in patients with unstable fracture and osteoporosis [10–14]. Moreover, ischiofemoral impingement, pseudoaneurysm of the femoral artery, and femoral nerve palsy caused by the migration of the LT-fragment following intramedullary fixation have also been reported [15–17]. Therefore, it is necessary to identify the factors associated with the union of the LT-fragment since it might affect the clinical outcomes in the treatment of intertrochanteric fractures.

However, there has been little research concerning the union of the LT-fragment following intramedullary fixation of intertrochanteric fractures. To the best of the authors’ knowledge, there have been no methods reported to evaluate the risks associated with the union of the LT-fragment, and the determination of such risk factors could help surgeons to decide when the LT-fragment needs to be refixed. Moreover, whether nonunion of the LT-fragment influences hip function and postoperative complications remains controversial.

Therefore, this study aimed to investigate the relationship between the distance of the displacement and the union of the LT-fragment and to evaluate the influence of nonunion of the LT-fragment on hip function and postoperative complications.

## Methods

### Study design and patients

This retrospective study included patients with intertrochanteric fractures and displaced LT treated with intramedullary fixation at the Department of Orthopedics, Wenzhou Hospital of Integrated Traditional Chinese and Western Medicine, from June 2015 to July 2017. The study was approved by the Institutional Review Board of Wenzhou Hospital of Integrated Traditional Chinese and Western Medicine. The requirement for individual informed consent was waived by the Board because of the retrospective nature of the study.

The inclusion criteria were 1) intertrochanteric fractures with LT-fragment, 2) treatment with a proximal intramedullary nail, 3) complete displacement of the LT-fragment, 4) ≥ 65 years of age, and 5) a minimum of 1 year of follow-up. The exclusion criteria were 1) previous hip or femur surgery on both sides, 2) malformation of the hip or femur on both sides, 3) old or non-osteoporotic pathologic fractures, 4) combined subtrochanteric fractures, 5) fixed LT-fragment, 6) small or comminuted fracture of the LT-fragment, 7) walking disability before the injury, 8) multiple fractures or open fractures.

All procedures were performed by the same team of senior surgeons at our institution. Intramedullary nail (PFNA or Gamma3, Sanatmetal, Hungray) was used for a standard implant.

### Data collection and definition

Patient medical records and digital radiographs were reviewed for each case. Patient age, sex, fracture side, bone mineral density (BMD), the Arbeitsgemeinschaft fur Osteosynthesfragen (AO) Foundation and Orthopedic Trauma Association (AO/OTA) classification of fractures [18], fracture reduction quality, follow-up, the union of intertrochanteric fractures, and the presence of LT-fragment were recorded. Fracture reduction quality was classified into three grades based on a method developed by Baumgaertner et al. [19]. The patients were retrospectively divided into two groups: those with union and those with nonunion of the LT-fragment, determined from a plain film radiograph at a minimum of 1 year of follow-up. The LT union were considered that X-rays clearly demonstrated bone bridging across the fracture, and fracture line blurred or disappeared.

The displacement of the LT-fragment was assessed on the anteroposterior (AP) view X-ray of both hip at 1-day postoperative. The height of healthy side LT > 20.8 mm or < 44.4 mm and the width > 4.4 mm or < 12.3 mm, which means external or internal rotation of the femur is less than 20° [20], as considered a relatively standard AP view X-ray for measurement. The vertical displacement (A) was defined as the vertical distance from the tip of the LT to the horizontal line of the acetabular apex on both sides. The horizontal displacement (B) was defined as the vertical distance from the tip of the LT to the edge of a line drawn along the lateral border of the femoral cortex. The vertical and horizontal distances of the healthy side also were measured as (A1 and B1). The displacement ratio was calculated according to the formula |(A-A1) |/A1 +|(B-B1) |/B1. Grouping and measurement were performed by two senior surgeons by reading radiographs on Picture Archiving and Communication System (PACS). If there is a discrepancy, another senior surgeon should be consulted.

The hip function, which was assessed using the Harris Hip Score (HHS), and postoperative complications, which included mechanical complications, cut-out, malunion/loss of reduction, excessive lateral migration of the blade (which had been defined as lateral migration of greater than or equal to 10 mm described by Liu et al. [21]), and other complications, were also evaluated at the final follow-up.

### Statistical analysis

Statistical analyses were performed with SPSS 16.0 (SPSS, Chicago, USA). The continuous data were presented as mean ± standard deviation and analyzed using Student’s t-test. The mean differences and the 95% confidence intervals (CIs) were calculated. Categorical data were presented as n (%) and analyzed using the chi-square or Fisher’s exact test. We compared the |(A-A1) |/A1 +|(B-B1) |/B1 ratios to the critical value to evaluate if a relationship might exist between the distance of displacement and the union of the LT-fragment. *P*-values < 0.05 were considered statistically significant.

The receiver operating characteristics (ROC) curve, which is defined as a plot of test sensitivity as the y-axis vs. its 1-specificity or false positive rate (FPR) as the x-axis, is an effective method of evaluating the performance of diagnostic tests. The area under the ROC curve (AUC) is a measure of the overall performance of a diagnostic test and is interpreted as the average value of sensitivity for all possible values of specificity [22]. MedCalc 11.4 (MedCalc Software bvba, Ostend, Belgium) was used to calculate the critical value using a ROC curve.

## Results

### Characteristics of the patients

**Fig. 1:**
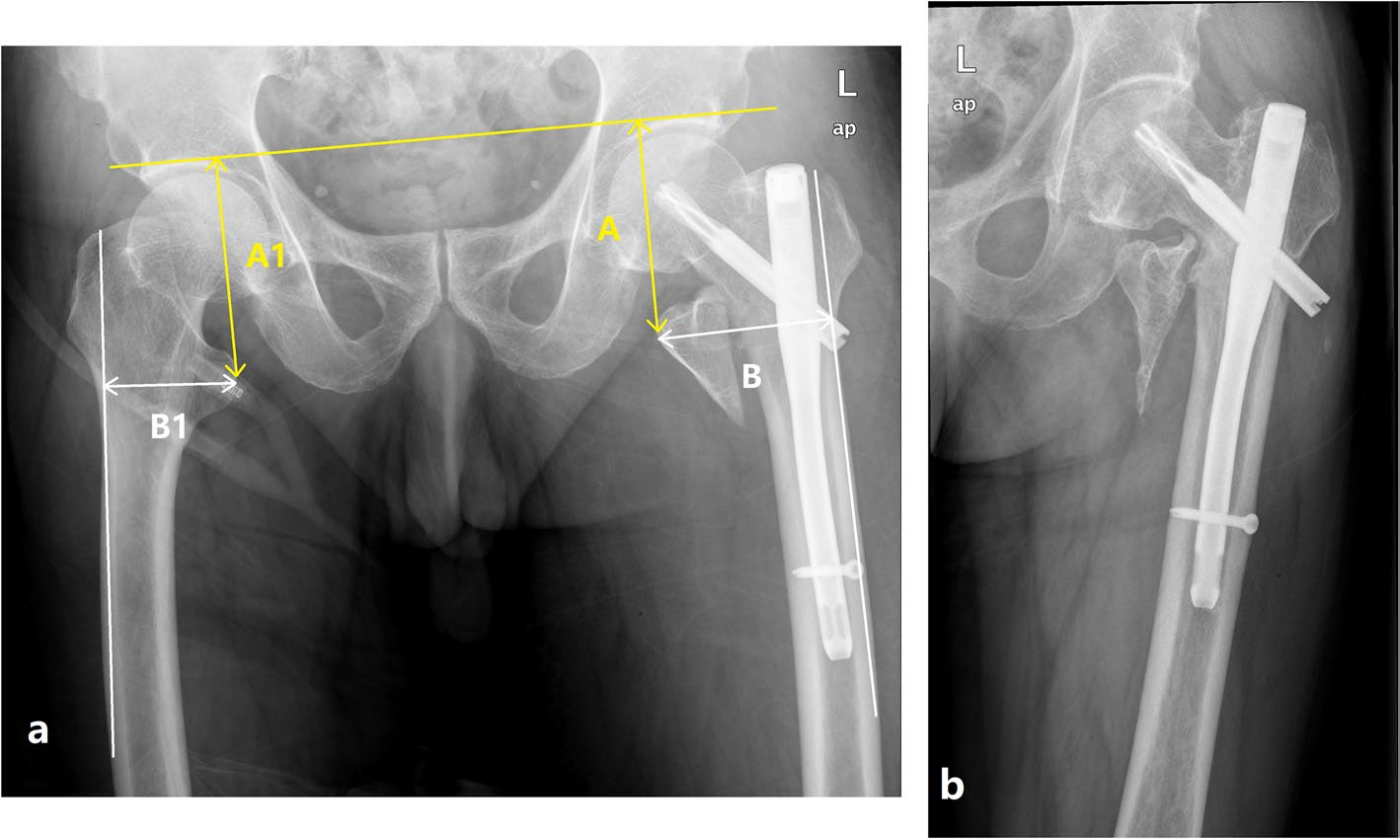
**(a)** A 74-year-old male patient with displacement of the lesser trochanter (LT) following intramedullary fixation of intertrochanteric fracture and the ratio of |(A-A1) |/A1 +| (B-B1) |/ B1 is 0.37. A was defined as the vertical distance from the tip of LT to the horizontal line of the acetabular apex on both sides. The horizontal displacement B was defined as the vertical distance from the tip of LT to the edge of a line drawn along the lateral border of the femoral cortex. The vertical and horizontal distance of the healthy side was considered as A1 and B1. L in figure means left side. **(b)** At 1-year follow-up, the LT-fragment showed nonunion, and the patient presented with excessive lateral migration of the blade and hip varus. L means left side

**Fig. 2:**
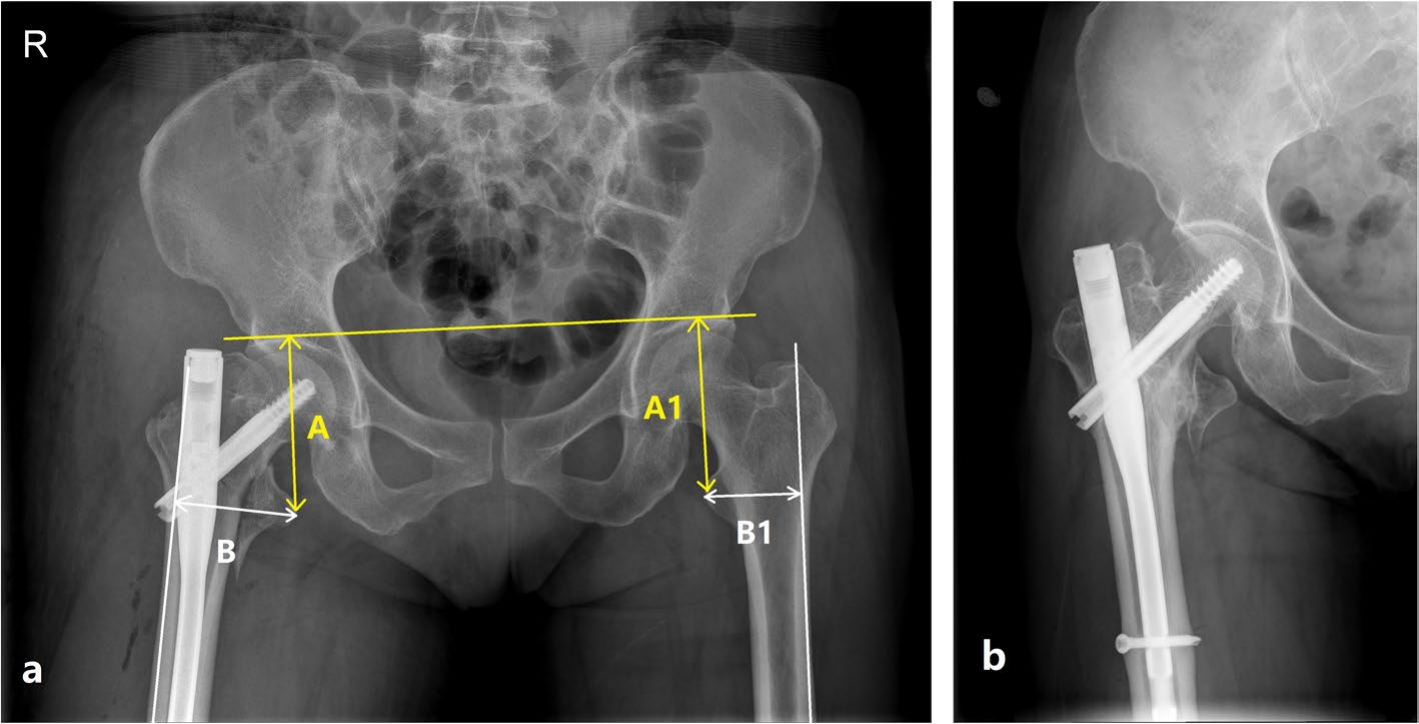
**(a)** A 68-year-old female patient with displacement of the lesser trochanter (LT) following intramedullary fixation of intertrochanteric fracture and the ratio of |(A-A1) |/A1 +|(B-B1) |/B1 is 0.23. **(b)** At 1-year follow-up, the LT-fragment showed union. R means right side

Fifty-three patients with trochanteric fractures and displaced LT were eligible for this study. The patients were divided into two groups: those with nonunion (Fig. 1) and union (Fig. 2) of the LT-fragment at a minimum 1-year follow-up. There were no statistically significant differences in sex, age, BMD, fracture side, fracture type, follow-up time, and union of intertrochanteric fractures between the two groups (all *P* > 0.05) (Table 1). All patients sustained their injury due to a simple fall. According to the AO/OTA classification, there were eleven A1 (20.8%), thirty-six A2 (67.9%) and sex A3 fractures (11.3%). Over 85% of the patients achieved good or acceptable fracture reduction. Fifty-two (98.1%) patients had an uneventful union of the intertrochanteric fractures. Only one (1.9%) patient had a further hemiarthroplasty because of a cut-out.

**Table 1:** Characteristics of the patients

Characteristics	Union group (*n* = 31)	Nonunion group (*n* = 22)	Mean difference, 95%CI	P
Sex				
Female	20 (65%)	16 (73%)	0.7 (0.21; 2.24)	0.528
Male	11 (35%)	6 (27%)	-	
Age, year	74.2 ± 7.0	73.1 ± 7.0	1.1 (-2.41; 4.7)	0.519
BMD (T scores)	-2.01 ± 0.72	-1.96 ± 0.72	0(-0.48;0.37)	0.807
AO/OTA classification				
A1	7 (23%)	4 (18%)	-	0.813
A2	20 (64%)	16 (73%)	-	
A3	4 (13%)	2 (9%)	-	
Fracture side				
Left	21 (68%)	13 (59%)	1.5, (0.47;4.53)	0.518
Right	10 (32%)	9 (41%)	-	
Reduction quality				
Good	20 (65%)	15 (68%)	-	0.957
Acceptable	6 (19%)	4 (18%)	-	
Poor	5 (16%)	3 (14%)	-	
Follow-up, months	14.7 ± 2.2	14.3 ± 2.2	0 (-0.82;1.54)	0.545
Union of intertrochanteic fractures				
Union	31 (100%)	21 (95%)	1 (0.96;1.15)	0.415
Nonunion	0	1 (5%)		

Data are expressed as mean ± standard deviation (SD) or n (%)

*BMD* Bone mineral density, *AO/OTA* Arbeitsgemeinschaft für Osteosynthesefragen Foundation and Orthopedic Trauma Association

### Hip function and postoperative complications at the final follow-up

Although HHS score was slightly lower in the nonunion group, no significant difference was observed in HHS score between two groups (*P* > 0.05). The rate of postoperative complications in the nonunion group was significantly higher than in the union group (59% vs. 29%, *P* = 0.047), especially for mechanical complications (45% vs. 6%, *P* = 0.001) (Table 2). In the nonunion group, there were 10 mechanical complications in seven patients, including one cut-out, five malunion/loss of reduction, and four excessive lateral migration of the blade. In the union group, there were two complications in two patients, including one malunion/loss of reduction and one excessive lateral migration of the blade.

**Table 2:** Hip function and postoperative complications at final follow-up

Characteristics	Union group (*n* = 31)	Nonunion group (*n* = 22)	Mean difference, 95%CI	P
Harris hip score	76.4 ± 9.1	68.8 ± 15.4	6.6 (-0.17; 13.36)	0.056
Pain	40.5 ± 1.4	39.5 ± 3.3	1.1 (-0.25; 2.38)	0.111
Functional disability	35.1 ± 3.0	35.6 ± 2.1	-0.6 (-2.09; 0.94)	0.452
Deformity	3.6 ± 0.6	3.6 ± 0.7	0 (-0.37; 0.36)	0.954
ROM	4.0 ± 0.6	4.0 ± 0.7	0 (-0.37; 0.31)	0.85
Postoperative complications^a^	9 (29%)	13 (59%)	0.3 (0.09; 0.9)	**0.047**
Mechanical complications	2 (6%)	10 (45%)	0.1 (0.02; 0.44)	**0.001**
**Cut-out**	0	1 (5%)	1 (0.96; 1.15)	
**Malunion/Loss of reduction**	1 (3%)	5 (23%)	0.1 (0.01; 1.05)	
**Lateral migration of blade**	1 (3%)	4 (18%)	0.2 (0.02; 1.45)	
Other complications	8 (26%)	5 (23%)	1.2 (0.33; 4.26)	0.797
Local soft tissue/wound	2 (6%)	1 (5%)	1.5 (0.12; 17.04)	
Systemic/rest of the body	6 (19%)	4 (8%)	1.1 (0.23; 4.4)	

Data are expressed as mean ± standard deviation (SD) or n (%), and data in bold indicate significant differences

*ROM* Range of motion

^a^The same patient can contribute to more than one category

### LT-fragment displacement at 1 day after surgery

Significant differences were found in the horizontal displacement of the LT-fragment (*P* = 0.027) and the displacement ratios (*P* < 0.001) between the two groups (Table 3). The proportion of cases with a displacement ratio > 0.35 was significantly higher in the nonunion group than in the union group (91% vs. 16%, *P* < 0.001). In addition, in patients with the ratios ≤ 0.35, only two of 28 patients showed nonunion of the LT fragment.

**Table 3:** The measurements on plain film radiograph at 1-Day postoperative

Characteristics	Union group (*n* = 31)	Nonunion group (*n* = 22)	Mean difference, 95%CI	P
Vertical displacement (A, mm)	76.4 ± 9.1	69.8 ± 15.4	6.6 (-0.17–0.88)	0.056
Horizontal displacement (B, mm)	59.9 ± 8.1	64.8 ± 7.7	-4.9 (-9.22–0.58)	**0.027**
Vertical distance of healthy side (A1, mm)	82.7 ± 8.7	80.7 ± 5.0	2 (-2.13–6.19)	0.291
Horizontal distance of healthy side (B1, mm)	51.5 ± 7.4	48.3 ± 4.5	3.2 (-0.35–6.75)	0.076
The ratio of displacement (|(A-A1) |/A1 +|(B-B1) |/B1)	0.3 ± 0.1	0.5 ± 0.2	-0.3 (-0.34–0.17)	** < 0.001**

Data are expressed as mean ± standard deviation (SD), and data in bold indicate significant differences

### ROC curve analysis

**Fig. 3:**
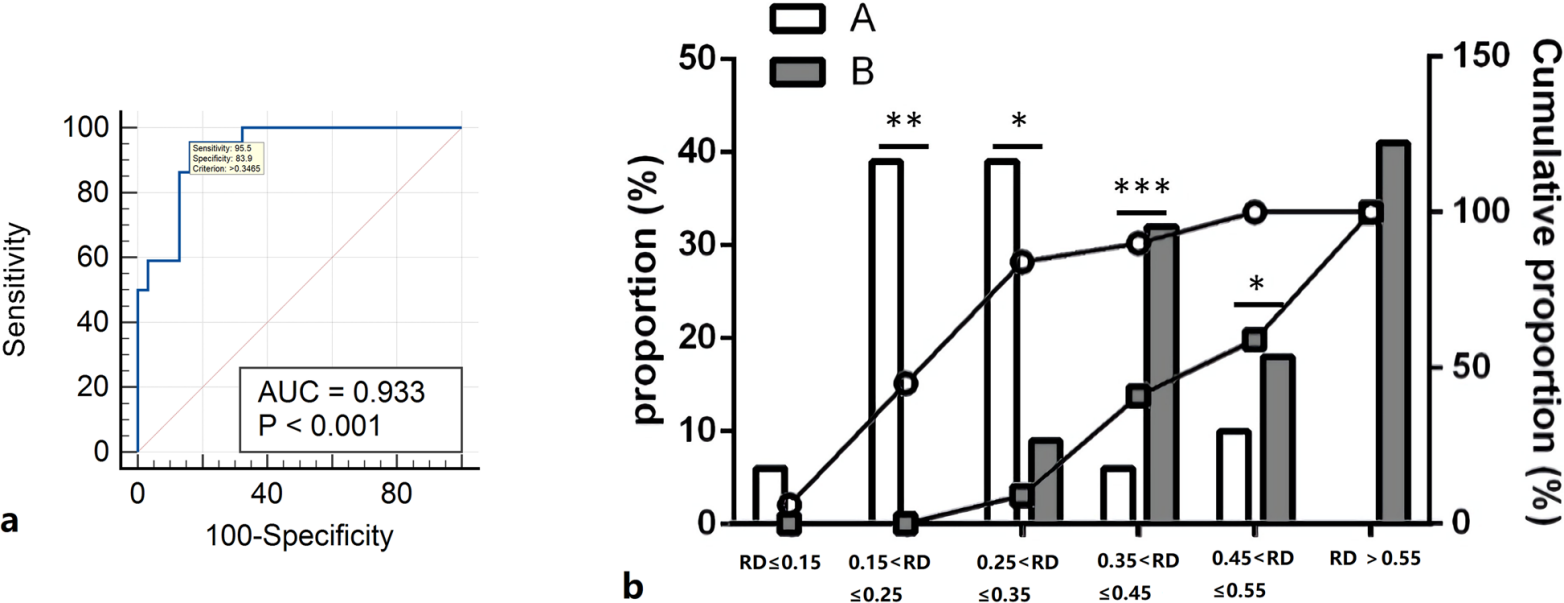
**(a)** The ROC curve reflects the relationship between the true positive rate (sensitivity) and false positive rate (1-specificity). The area beneath the ROC curve reflects the likelihood of causing nonunion of LT. According to the ROC curve in our study, the area under the ROC curve was 0.933. After comprehensive consideration of sensitivity and specificity, we defined 0.35 as the critical value. **(b)** The distribution and cumulative curve of displacement ratios. *, ** and *** mean *P* < 0.05, *P* < 0.005 and *P* < 0.001 respectively. RD: Ratio of Displacement

According to the ROC curve (Fig. 3 a), the AUC was 0.933, indicating that the displacement ratio is a reliable predictor of the union of the LT-fragment. The Youden index showed that the best critical value was 0.35. Patients with a displacement ratio over 0.35 were more likely to have nonunion of the LT-fragment (Fig. 3 b).

## Discussion

Displacement of the LT is not uncommon after the management of intertrochanteric femoral fractures. The influence of nonunion of the LT-fragment on clinical outcomes remains controversial. This study aimed to investigate the relationship between the displacement distance and union of the LT-fragment and evaluate the influence of LT-fragment nonunion on hip function and complications. The present study indicated that a significant relationship exists between the distance of displacement and the union of the LT-fragment after the treatment of intertrochanteric fractures with intramedullary fixation. Our results suggest that union cases which had less displacement were inherently more stable with less complications, and nonunion cases had more displacement were inherently more unstable with more complications.

Intertrochanteric fractures commonly occur in older adults, and more than 50% of the cases are associated with a complete fracture of the LT [23]. Several studies demonstrated that the LT, as a major part of posteromedial construction, plays a significant role in the stability of unstable osteoporotic intertrochanteric fractures [10–13]. Moreover, a biomechanical study showed that the stability decreased gradually with the increasing size of the LT-fragment [10]. A retrospective review of 111 intertrochanteric fractures with displaced LT-fragment treated with intramedullary nailing showed that a severely displaced LT-fragment increased postoperative complications and postoperative pain [24]. Speculatively, the union of the LT-fragment might be crucial for the clinical outcome of intertrochanteric fractures. Still, despite a thorough literature review, no methods have been reported to evaluate the risk of union of the LT-fragment. To the best of the authors’ knowledge, this is the first quantitative imaging analysis of the risk of nonunion of the LT-fragment in treating intertrochanteric fractures. This study can help surgeons decide for LT-fragment refixation by a ratio-based measurement of the displacement. The authors propose that the risks of union can be evaluated using the anteroposterior radiographs of both hips intraoperatively, measuring the ratios of displacement, then comparing them to the critical value of 0.35. If the ratio is > 0.35, reduction and refixation of the LT fragment need to be considered. However, since refixation of LT can cause additional trauma which may be fatal for elderly patients, the treatment plan should be individualized after carefully weighing the patients’ possible benefits and risks.

The findings confirmed that postoperative complications were higher in patients with nonunion of the LT-fragment compared to those with union, as supported by Sun et al. [24]. In this study, the incidence of postoperative complications in the nonunion group was significantly higher than in the union group, especially for mechanical complications. The importance of the LT-fragment in the stability of proximal femur fractures has been adequately confirmed by biomechanical studies [11, 13]. The union of the LT-fragment, which represents the integrity of the medial cortex, might be strongly associated with the stability of intertrochanteric fractures. Thus, nonunion of the LT-fragment would lead to internal fixation failure, malunion, or loss of reduction. These reasons might help explain the postoperative mechanical complications that often occur in patients with a nonunion of the LT-fragment.

The present study findings are supported by previous research showing that the displacement of the LT-fragment following intramedullary fixation of intertrochanteric fractures does not affect the hip function. Indeed, Liu et al. [9] compared intertrochanteric fracture patients with and without preoperative LT integrity retrospectively and reported no significant difference between the two groups, supporting the present study. Clinical studies reported no relationship between LT dislocation and strength of hip flexion [24, 25].

The strengths of this study include the simple and accurate measurement by PACS, the same conditions and interventions for the two groups, and the similar patient demographics that allowed for unbiased group comparisons. Besides, considering the individual differences among patients, the distances of vertical and horizontal displacements were compared with the healthy side on the same patient, and the ratio was used as a comparison parameter of displacement.

This study had several limitations. Firstly, since this was a retrospective study, there were some missing information, incomplete medical records and inconsistent radiographs quality for a small part of patients. Secondly, although the vertical and horizontal displacements were assessed, the size and rotation of the LT-fragment were difficult to measure on plain film, which might influence the results. Thirdly, due to pain, fear, and other negative factors on 1-day post-operation, internal and external rotation of the lower limbs might occur during the X-ray shooting, which makes it hard to take a very standard AP view radiography. Although relatively standard AP view radiography were used in our study, it also might affect the accuracy of measurement. Furthermore, the assessment is difficult to be performed intraoperatively as it depends on an AP view X-ray of both hips taken by Digital Radiography(DR). Lastly, the sample size enrolled in our study was relatively small and the follow-up was relatively short. Large-sized prospective randomized trials with long follow-up were needed. Though there are some potential sources of bias in this study, it has raised concerns about the potential impact of the displaced LT. Our study indicates that LT union cases were inherently more stable with less displacement and LT nonunion cases were inherently more unstable with more displacement.

## Conclusions

In conclusion, the displacement ratio might be a reliable predictor of LT-fragment union. The nonunion of the LT-fragment did not influence hip function but might increase the incidence of postoperative complications. Refixation of the LT-fragment might be considered if the displacement ratio exceeds 0.35, but additional studies are needed for confirmation and validation.

## Data Availability

All data generated or analysed during this study are included in this published article.

## Abbreviations

LT: Lesser trochanter
ROC: Receiver operating characteristic
BMD: Bone mineral density
CIs: Confidence intervals
FPR: False positive rate
AUC: Area under the ROC curve
DHS: Dynamic hip screw
RD: Ratio of displacement
PACS: Picture archiving and communication system
AP: Anteroposterior
